# Rat Facial Nerve Regeneration with Human Immature Dental Pulp Stem Cells

**DOI:** 10.1177/0963689719854446

**Published:** 2019-08-28

**Authors:** Daniel Martinez Saez, Robson Tetsuo Sasaki, Daniel de Oliveira Martins, Marucia Chacur, Irina Kerkis, Marcelo Cavenaghi Pereira da Silva

**Affiliations:** 1 Department of Morphology and Genetics, Universidade Federal de São Paulo, São Paulo, Brazil; 2 Departament of Anatomy, Institute of Biomedical Sciences – Universidade de São Paulo, São Paulo, Brazil; 3 Department of Genetics, Instituto Butantan, São Paulo, Brazil

**Keywords:** facial nerve, nerve injuries, stem cells, deciduous teeth

## Abstract

Facial paralysis can result in severe implications for the patients. However, stem cell biology has become an important field in regenerative medicine since the discovery and characterization of mesenchymal stem cells. Our aim was to evaluate the regeneration after facial nerve crush injury and application of human immature dental pulp stem cells (iDPSC). For this study 70 Wistar rats underwent a unilateral facial nerve crush injury and were divided into two groups: Group I (GI): Crushed; Group II (GII): Crushed and iDPSC, and distributed into study periods of 3, 7, 14, 21, and 42 postoperative days. Facial nerve regeneration was analyzed via functional recovery of whisker movement, histomorphometric analysis, and immunoblotting assay. The results show that GII had complete functional recovery at 14 days, while GI recovered after 42 days. Also, regarding the facial nerve trunk, GII presented histological improvement, evidencing better axonal and structural organization of the myelin sheath, and exhibited statistically higher values for the outer and inner perimeters and g-ratio. Nevertheless, GI exhibited statistically higher values for the thickness of myelin sheath. In the buccal branch, no differences were observed for all parameters between groups. At 42 days, both groups GI and GII were close to the levels observed for the control group. Concerning nerve growth factor expression, GII exhibited statistically greater values (*p* < 0.05) compared with the control group at 7 days. In summary, a single injection of human iDPSC promoted a positive effect on regeneration of the facial nerve trunk after 14 days and provided an alternative to support regeneration following peripheral nerve injury.

## Introduction

The facial nerve provides mobility of mimicry and neck muscles. Because of its long extratemporal segment upon emerging from the stylomastoid foramen and superficial location, it is commonly affected by traumatic lesions, which are possible causes of peripheral facial palsy^1,2^.

After injury, there is reduction or loss of muscle mobility in the affected hemiface, causing facial asymmetry^3^. Thus, functional disorders of the facial nerve are not restricted to physiological alterations, but are accompanied by disorders in speech and mastication, as well as esthetic and psychological involvement due to significant changes in self-esteem and social living^2,4^.

The successful regeneration of peripheral nerve lesions depends on the support of Schwann cells (SC), which participate in the phagocytosis of axons and myelin debris and later in the myelinization of newly formed axonal fibers, besides production of neurotrophic factors, including neural growth factor (NGF), adhesion molecules (CAMs), cytokines, and/or junctional structures^5–9^, contributing to regularization and modulation of neuronal survival^8,10^.

Therefore, the levels of neurotrophic factors differ from those found in non-damaged tissues^11,12^. The expression of neurotrophic factors is significantly increased in the early lesion, returning to normal levels after axonal regeneration^13,14^.

However, the proliferation of endogenous SC is insufficient for neural regeneration, requiring exogenous complementation by transplantation of SC^6,15,16^, administration of trophic factors^8,10^, or utilization of stem cells to induce regeneration of peripheral nerves^17–20^.

Several types of stem cells have been identified and isolated from oral cavity tissues^21–27^. Deciduous teeth stem cell populations (DTSCs) have demonstrated great potential of proliferation and population duplications, presenting as multipotent stem cells which, under chemically defined culture conditions, are able to proliferate rapidly into different cell lineages such as bone, cartilage, muscle, and neural cells^22,27,28^.

Due to their origin from the neural crest, human immature dental pulp stem cells (iDPSC) present neurogenic potential in vivo by expressing a variety of neural cell markers, including nestin, β3-tubulin, glutamic acid decarboxylase (GAD), neuronal nuclei, glial fibrillary acid protein (GFAP), neurofilament M (NFM), and 2’,3’-nucleotide 3’-phosphodiesterase^9,22,28^. They also express neurotrophins, including NGF, which promote migration and proliferation of SC to the target tissue, favoring a regenerative microenvironment^9,29,30^.

Considering the regenerative potential of DTSCs for peripheral nerve injuries, this study evaluated the regeneration of the facial nerve of Wistar rats after crush injury of the facial nerve trunk and application of iDPSC, by quantitative and qualitative methods at different postoperative periods.

## Materials and Methods

### Study Groups

All experimental procedures and protocols involving animals, including animal housing conditions, surgical procedures, and postoperative care, were approved by the Institutional Review Board on Animal Studies of the Universidade Federal de São Paulo.

This study was conducted on 70 Wistar rats with average weight between 280 and 300 g. They were housed in individual cages for 7 days for acclimatization at 23°C room temperature with a 12:12 light–dark cycle, considering light from 7 am to 7 pm. For transmission electron microscopy (TEM) analysis, 30 animals were randomly selected and divided into two study groups (*n* = 15) and for immunoblotting assay, 40 animals were selected and divided into two study groups (*n* = 20). The animals underwent a unilateral facial nerve crush injury (left hemiface) and were divided into two groups: Group GI (positive control): animals submitted to injury; and Group GII (experimental group): animals submitted to injury and treatment with human iDPSC. The contralateral side (right hemiface) of GI animals served as a group control: GC (negative group). The facial nerve trunks were collected and are represented by the letter T (GIT or GIIT), and the buccal branches of the facial nerve are represented by the letter R (GIR or GIIR).

### Cell Culture

The use of human stem cells was approved by the Institutional Ethical Committee of the Universidade Federal de São Paulo. The normal exfoliated human deciduous teeth of 5- to 7-year-old children (10 patients) were extracted under local anesthetic at a dental clinic after informed consent was obtained. Informed consent was written and provided by the participants and/or provided by a legally authorized representative.

Dental pulp was pulled out with a barbed Nerv-broach (dental instrument), washed twice with sterile phosphate buffered saline (PBS; 0.01 M, pH = 7.4) supplemented with antibiotics (100 U/ml penicillin and 100 g/ml streptomycin), and transferred (with minimal dissection) into 35-mm Petri dishes (Corning^®^, New York, NY, USA) with Dulbecco’s modified Eagle’s medium (DMEM)/Ham’s F12 (1:1, Invitrogen, Carlsbad, CA, USA) supplemented with 15% fetal bovine serum (FBS) (HyClone, Logan, UT, USA), 100 U/ml penicillin, 100 g/ml streptomycin, 2 mM L-glutamine, and 2 mM nonessential amino acids. Tissue explant of dental pulp was used to isolate immature stem cells. Isolation, cultivation, and characterization of this population of mesenchymal stem cells was obtained in accordance with the Kerkis method^27^.

Early passages of obtained human iDPSC with normal karyotype were grown in DMEM supplemented with 10% FBS, 100 U/ml penicillin, 100 g/ml streptomycin, and 2 mM L-glutamine (Invitrogen). All cultures were incubated at 37°C in 5% CO_2_ and high humidity.

### Surgical Procedures and Euthanasia

Under anesthesia with 2% xylazine (0.025 ml/100 g) and 10% ketamine hydrochloride (0.05 ml/100 g), a retroauricular skin incision was made. Then, the facial nerve trunk, distal to the stylomastoid foramen, was strongly crushed with forceps perpendicular to the longitudinal axis of the nerve. For consistency in amount of pressure applied during the crush, the same researcher crushed all nerves in this study and used the third ratchet of the ultra-fine forceps for 30 s. After surgery, animals in GI received application of culture medium (4 μl of DMEM) and GII received culture medium associated with human iDPSC (5×10^5^ cells in 4 μl DMEM), applied immediately after the crush injury. Then, the skin incision was sutured with 3.0 Vicryl suture.

The animals were placed into individual cages to recover and maintained under analgesics (200 mg/l Paracetamol, Medley, São Paulo, Brazil) diluted in water for 3 days, and under antibiotics (Enrofloxacin 2.5 mg/kg, Bayer, São Paulo, Brazil), in a single dose.

The success of surgery was confirmed after the recovery of the animals from anesthesia, assessing the loss of blinking reflex, position, and movement of vibrissae in the left hemiface.

The animals of all groups were euthanized at 3, 7, 14, 21, and 42 days after surgery with an intraperitoneally lethal dose of pentobarbital anesthetics.

### Behavioral Method of Whisker Movement

On the day of euthanasia, the movements of the vibrissae were videotaped using a high-definition video camera. A black background was placed between the whiskers and the floor, to improve visualization of the whisker movement. To ensure the reliability of facial nerve function, the video clips were independently analyzed by three examiners blind to treatment group, who were allowed to view the images on two occasions, with a 7-day interval between them. They observed two parameters, namely symmetric or asymmetric movement and positioning of vibrissae, comparing the left side (operated) and the right side (intact) of animals and assigning a numeric value according to the scale presented in Table 1.

**Table 1.** Scoring Scale for Whisker Movement Observation.

Score	Movement and Positioning
0	No motion
1	Minor trembling
2	Effective movement and posterior positioning
3	Effective movement and anterior positioning

### TEM Analysis

The collected facial nerve trunk and buccal branch were pre-fixated in 4% PFA solution and post-fixated in 1% osmium tetroxide, embedded and polymerized for 72 h at 60°C. Sections of 0.5 μm thickness were stained with toluidine, observed on Olympus BX41 (Olympus, Shinjuku, Japan) and images were acquired using Axiovision software (version 4.5). Ultra-thin 0.3-μm sections were collected on copper grids, contrasted in 5% uranyl acetate and 1% lead citrate, and examined on a transmission electron microscope JEOL JEM 1010 TEM 80 kV (JEOL Ltd, Akishima, Japan). Images of neural fibers were obtained at 2,500×, 20,000×, and 100,000× magnification for qualitative analysis, and morphometric parameters (outer perimeter of nerve fiber; inner axonal perimeter; myelin sheath thickness and g-ratio) were measured on the software ImageJ (NIH image, Bethesda, MD, USA).

### Immunoblotting Assay

After homogenization of facial nerve trunk fragments and addition of 10% of volume used in a buffered 10% Triton X-100 solution for half an hour, the specimens were centrifuged at a crosshead speed of 12,000 rpm for 20 min at 4°C, and the quantity of protein was determined by the Bradford method^31^.

After quantification of total protein, the specimens were analyzed by SDS-PAGE electrophoresis. The materials were diluted in the same volume of buffered Tris/HCl 125 mM, pH 6.8, containing 2.5% (p/v) of SDS, 2.5% of 2-mercaptoethanol (2-ME), 4 mM of EDTA and 0.05% of bromophenol blue, followed by boiling in water bath for 5 min.

Sequentially, the specimens were applied on 10% polyacrylamide gel and submitted to electrophoresis at a continuous current of 120 V. After electrophoretic separation, the proteins were transferred to a nitrocellulose membrane (Millipore, 0.2-µm diameter) according to the technique described by Towbin and Gordon^32^. Antigens present in the nitrocellulose membrane were submitted to immunoenzymatic characterization. After blocking using skim milk Molico^®^ (Nestlé, São Paulo, Brazil) at 5% in Tris-Saline buffer (Tris 10 mM and NaCl 0.15 M, pH 7.5), for 1 h, the membranes were incubated with primary antibody NGF 1:1000 (Santa Cruz Biotechnology Inc., Dallas, TX, USA).

The antibody was diluted in a blocking solution for 18 h at 4°C. Following this, the membranes were rinsed with Tris-Saline and incubated for 2 h with the secondary antibody goat anti-rat (Invitrogen) labeled with peroxidase, diluted at 1:5,000 in a blocking solution. The excess was removed by additional rinsing with Tris-Saline. β-actin was used as internal control of the reaction (monoclonal mouse anti- β-actin [1:10,000], Sigma-Aldrich, St. Louis, MO, USA). The membranes were revealed using the chemiluminescence kit ECL (Amersham Biosciences Corporation, Piscataway, NJ, USA) and analyzed as to the optical density of labeled bands, using the software Scion Image (Scion Corporation, Frederick, MD, USA).

### Statistical Analysis

The results of functional recovery whisker movement were submitted to the Kappa concordance test. Generalized Linear Model GENLIN^®^ (IBM, New York, NY, USA) was used for quantitative analysis of TEM data, and data are expressed as mean and standard deviation (SD). The g-ratio was obtained by division between the axon diameter and nerve fiber diameter. These statistical analyses were performed using SPSS – version 23.0 (IBM). The results of immunoblotting assays were statistically analyzed on the software GraphPad Prism 5 (GraphPad Software, La Jolla, CA, USA) by one-way ANOVA and post-Tukey test. For all comparisons, a *p*-value less than 0.05 was considered significant.

## Results

### Functional Recovery of Whisker Movement

All animals lost whisker movement after crush injury. In addition, both groups exhibited no whisker movement at 3 postoperative days and only minor trembling of whiskers at day 7 (Table 2).

**Table 2.** Scores Obtained by Examiners from the Observation of the Whisker Movements of the Animals, for All Periods of the Study.

Periods (days)	Score - Whisker Movement					
	Examiner I		Examiner II		Examiner III	
	Group I	Group II	Group I	Group II	Group I	Group II
3	0	0	0	0	0	0
7	1	1	1	2	1	1
14	1	3	1	3	1	3
21	2	3	2	3	2	3
42	3	3	3	3	2	3

Animals in group GII presented complete functional recovery of vibrissae movement 2 weeks after treatment, but animals in group GI exhibited gradual functional recovery and complete functional recovery after 42 days.

The results of the examiners’ evaluations were submitted to the Kappa concordance test and the intra-examiner agreement analysis showed a general Kappa value of 0.812, which points to excellent agreement for each examiner.

### Nerve Morphological Analysis

**Figure 1.**
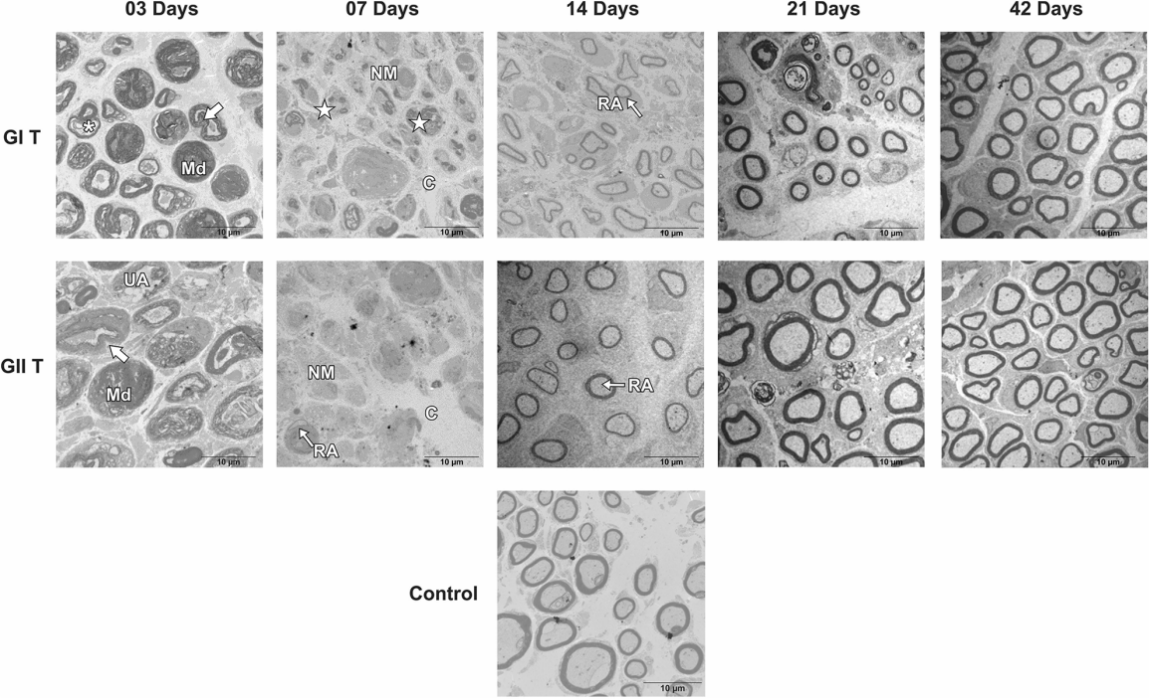
Cross-section TEM images of the facial nerve trunk for the GC, GI, and GII groups on 3, 7, 14, 21, and 42 days (2,500× magnification). (*) Myelin disruption; (Md) Myelin debris; (arrow) Myelin sheath infolding; (star) recent demyelination; (NM) Non-myelinated nerve fibers; (UA) Unmyelinated axon; (C) Connective tissue; (RA) Remyelinated axon. Scale Bar = 10 µm.

**Figure 2. fig2-0963689719854446:**
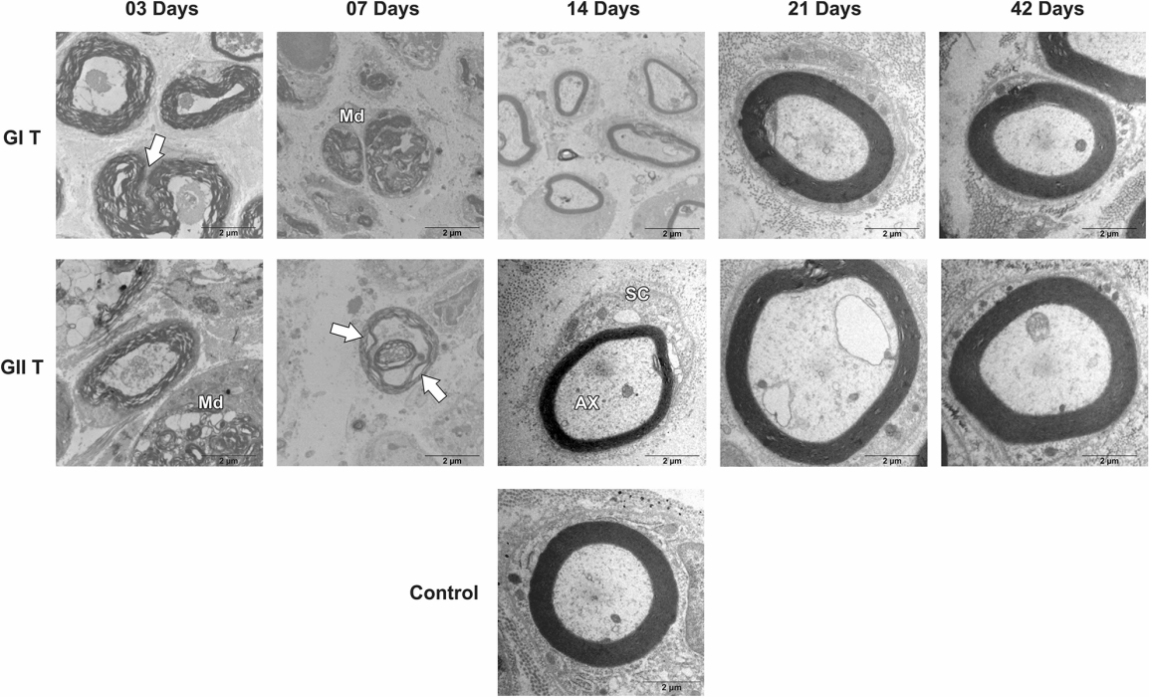
Cross-section TEM images of the facial nerve trunk for the GC, GI, and GII groups on 3, 7, 14, 21, and 42 days for ultra-structural analysis (20,000× magnification). (Md) Myelin debris; (arrow) Myelin sheath infolding; (Ax) Myelinated axon; (SC) Schwann cell. Scale Bar = 02 µm.

**Figure 3. fig3-0963689719854446:**
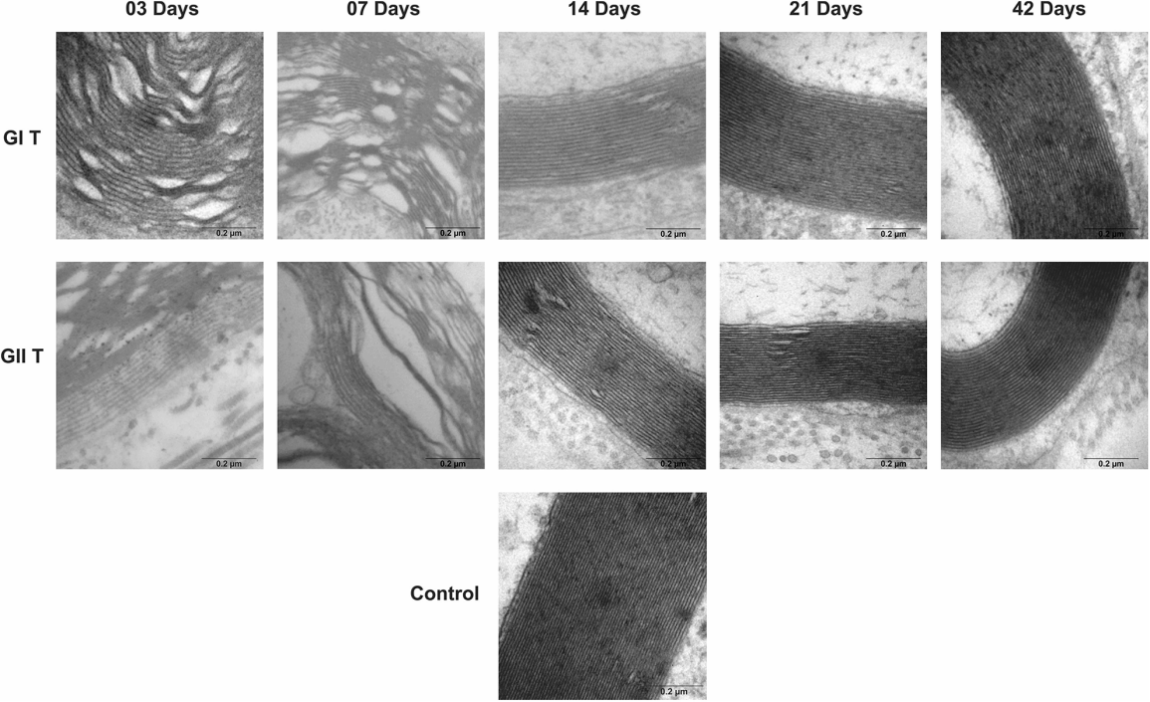
Cross-section TEM images of the facial nerve trunk for the GC, GI, and GII groups on 3, 7, 14, 21, and 42 days for ultra-structural analysis (100,000× magnification) of lamellae of the myelin sheath. Scale Bar = 0.2 µm.

**Figure 4. fig4-0963689719854446:**
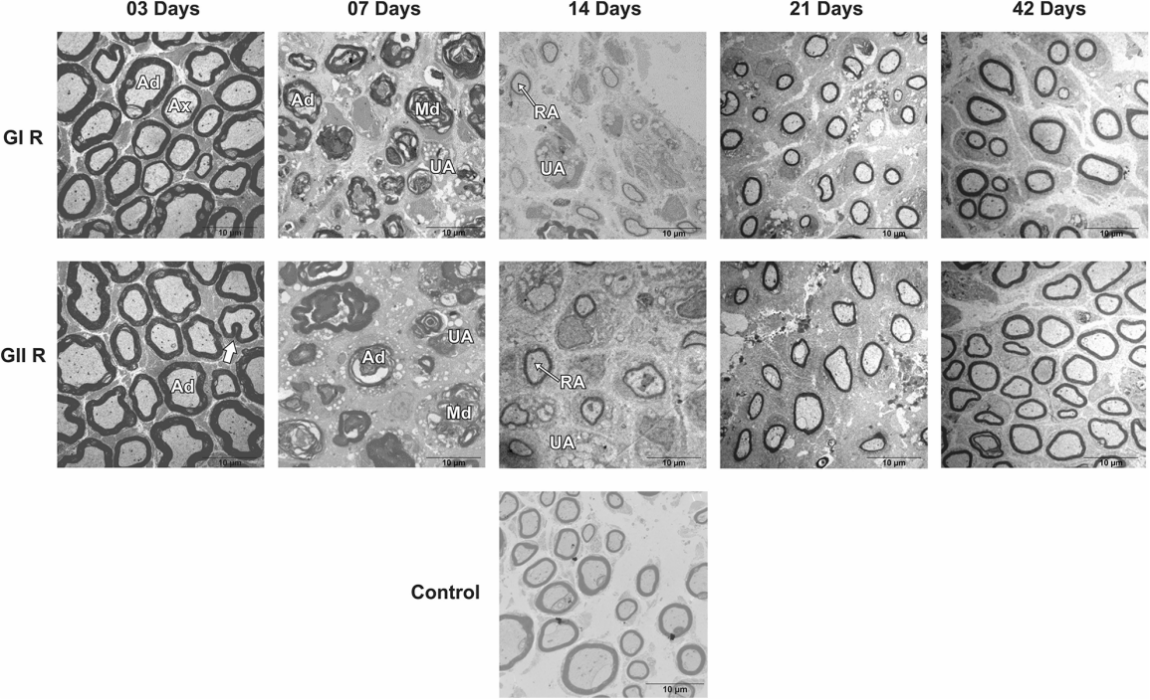
Cross-section TEM images of the buccal branch for the GC, GI, and GII groups on 3, 7, 14, 21, and 42 days (2,500× magnification). (Md) Myelin debris; (arrow) Myelin sheath infolding; (Ax) Myelinated axon; (UA) Unmyelinated axon; (Ad) axon degeneration; (RA) Remyelinated axon. Scale Bar = 10 µm.

**Figure 5. fig5-0963689719854446:**
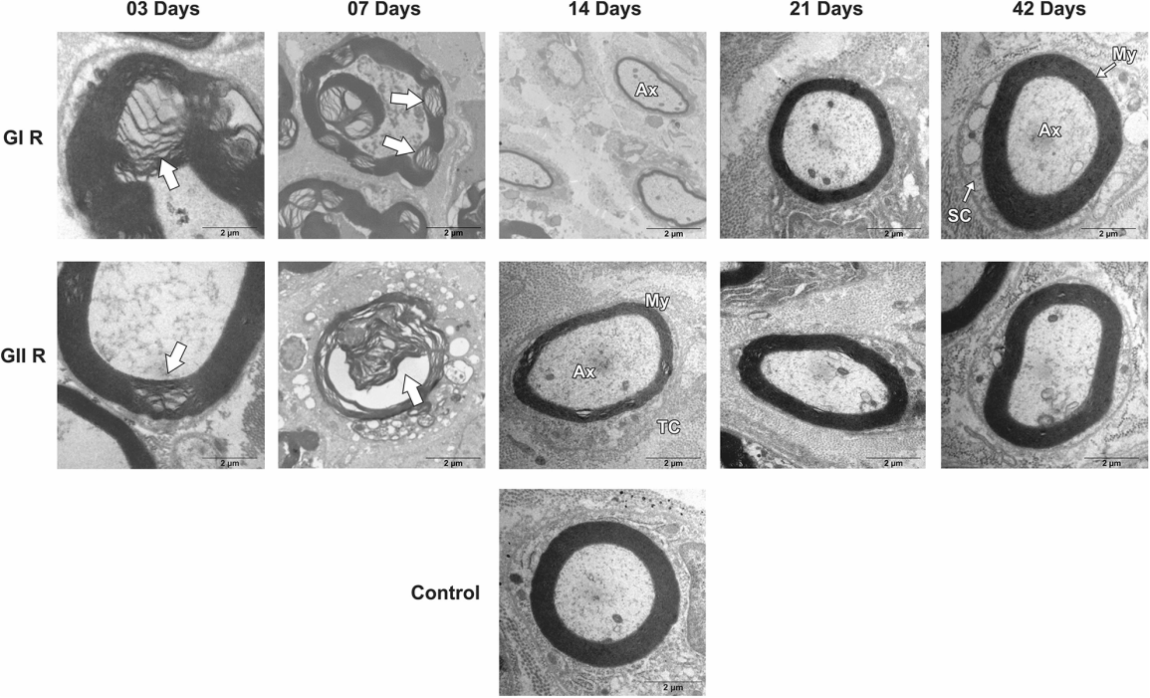
Cross-section TEM images of the buccal branch for the GC, GI, and GII groups on 3, 7, 14, 21, and 42 days for ultra-structural analysis (20,000× magnification). (arrow) Myelin sheath infolding; (Ax) Myelinated axon; (My) Myelin sheat; (C) Connective tissue; (SC) Schwann cell. Scale Bar = 02 µm.

Groups GI and GII did not reveal important differences in trunk and buccal branch at 3 and 7 days, but it was possible to observe the presence of axon degeneration, non-myelinated axons, and nerve fibers with recent demyelination and disorganization of myelin sheath lamellae at higher magnification (Figs 1 [Fig fig2-0963689719854446] [Fig fig3-0963689719854446] [Fig fig4-0963689719854446] [Fig fig5-0963689719854446]–6).

**Figure 6.**
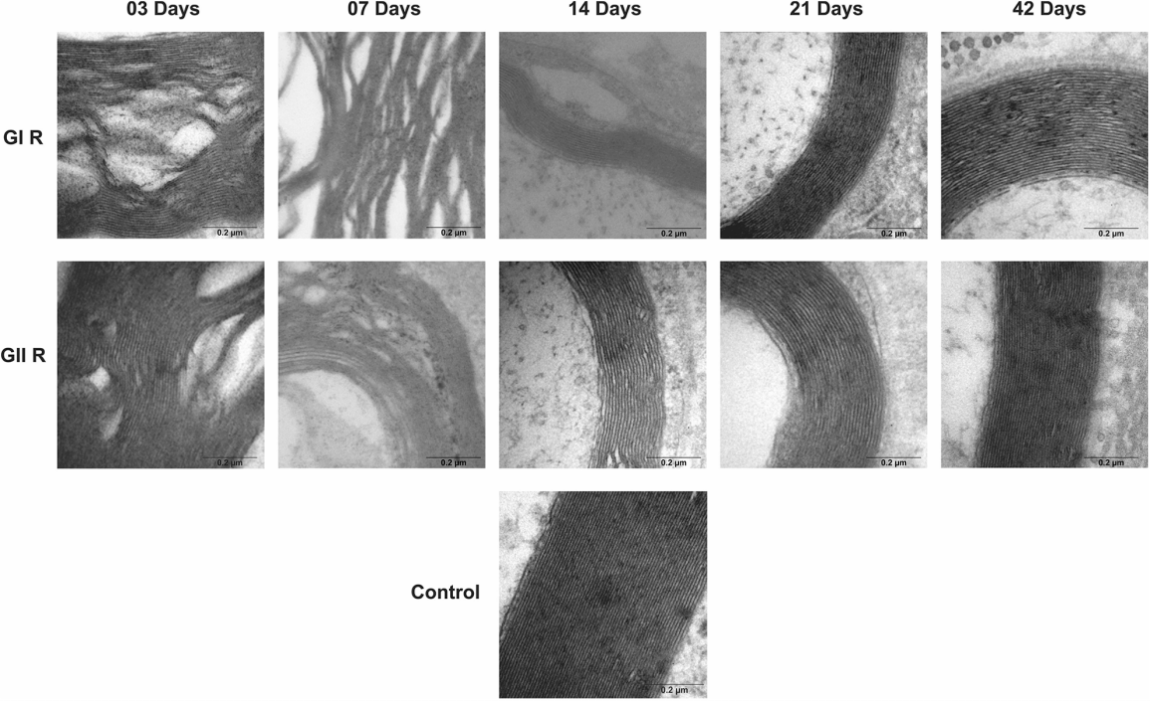
Cross-section TEM images of the buccal branch for the GC, GI, and GII groups on 3, 7, 14, 21, and 42 days for ultra-structural analysis (100,000× magnification) of lamellae of the myelin sheath. Scale Bar = 0.2 µm.

At 14 days, GIT and GIIT exhibited remyelinated axons (Fig. 1) but the axons of GIIT presented more regular morphology with rounded aspect and surrounding SC (Fig. 2), and exhibited lamellar organization (Fig. 3). In addition, compared with GIR, GIIR revealed more regular organization of the connective tissue (Figs 4 and 5) and myelin sheath lamellae at higher magnification (Fig. 6).

At 21 and 42 days, in trunk and buccal branch, groups GI and GII presented homogeneity of axons and revealed similar morphological aspect compared with GC (Figs 1 [Fig fig2-0963689719854446] [Fig fig3-0963689719854446] [Fig fig4-0963689719854446] [Fig fig5-0963689719854446]–6).

The mean thickness of the myelin sheath in the crushed group presented greater values compared with the cell transplantation group on postoperative days 3, 7, 14, 21, and 42, as shown in Table 3. Moreover, the mean outer and inner perimeters of axons in GIIT, at all periods, were statistically greater (*p* < 0.05) compared with the means of GIT.

**Table 3.** Mean Values of Outer Perimeter, Inner Perimeter and Thickness of the Myelin Sheath in Function of Timespan and Studied Groups.

		Facial Nerve Trunk (*n* = 30)				Buccal Branch (*n* = 30)			
		GIT (*n* = 15)		GIIT (*n* = 15)		GIR (*n* = 15)		GIIR (*n* = 15)	
		M	SD	M	SD	M	SD	M	SD
Outer Perimeter (µm^2^)	03 Days (*n* = 3)	38.17	2.95	50.76	3.83	40.24	3.04	41.22	2.96
	07 Days (*n* = 3)	17.96	2.16	23.89	2.74	18.94	2.29	19.40	2.34
	14 Days (*n* = 3)	12.21	0.65	16.23	1.01	12.87	0.87	13.18	0.87
	21 Days (*n* = 3)	12.82	0.68	17.04	0.93	13.51	0.77	13.84	0.78
	42 Days (*n* = 3)	21.58	1.13	28.70	1.53	22.75	1.26	23.31	1.33
Inner Perimeter (µm^2^)	03 Days (*n* = 3)	17.50	1.55	25.68	2.19	19.47	1.68	18.21	1.45
	07 Days (*n* = 3)	7.39	1.02	10.85	1.42	8.23	1.15	7.69	1.07
	14 Days (*n* = 3)	6.42	0.39	9.42	0.65	7.15	0.54	6.68	0.50
	21 Days (*n* = 3)	6.77	0.41	9.93	0.61	7.53	0.48	7.04	0.44
	42 Days (*n* = 3)	10.15	0.59	14.89	0.88	11.29	0.71	10.56	0.68
Thickness of the Myelin Sheath (µm)	03 Days (*n* = 3)	1.34	0.04	1.23	0.04	1.22	0.04	1.20	0.03
	07 Days (*n* = 3)	1.17	0.06	1.07	0.05	1.07	0.05	1.05	0.05
	14 Days (*n* = 3)	1.06	0.04	0.97	0.04	0.97	0.04	0.95	0.04
	21 Days (*n* = 3)	1.07	0.03	0.98	0.02	0.97	0.03	0.95	0.03
	42 Days (*n* = 3)	1.08	0.02	0.98	0.02	0.98	0.02	0.96	0.02

**Figure 7.**
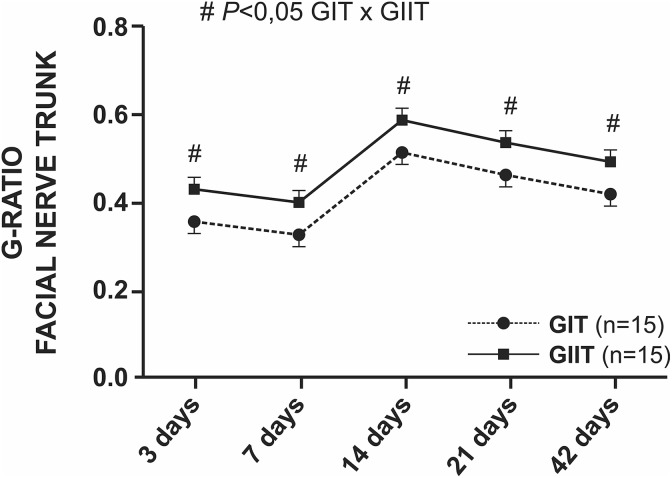
Statistical analysis of the g-ratios of the facial nerve trunk. The g-ratio was significantly improved in the GII group relative to the GI group (^#^ *p* < 0.05).

The g-ratio revealed reduction of values up to 7 days, followed by maximum value at 14 days and a decrease in the last 4 weeks for both groups. Group GIIT animals presented statistically greater values (*p* < 0.05) compared with group GIT (Fig. 7 and Supplementary Material I).

**Figure 8.**
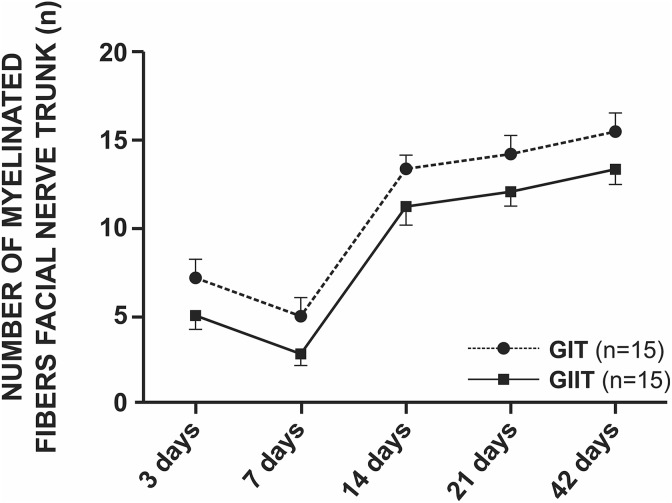
Statistical analysis of the number of myelinated fibers of the facial nerve trunk. There was no statistical difference between groups.

With regard to the number of myelinated fibers in the trunk, there was no statistical difference in the means between the studied groups, even though the means of GIT were greater compared with GIIT (Fig. 8 and Supplementary Material I). Here, it is possible to observe a decrease in the number of myelinated fibers until the seventh day, followed by a rapid increase at 14 days and then a gradual increase until 42 postoperative days.

**Figure 9.**
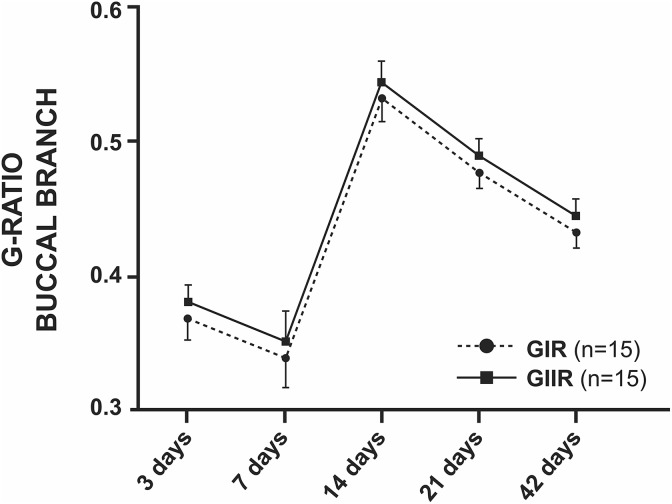
Statistical analysis of the g-ratios of the buccal branch. There was no statistical difference between groups.

**Figure 10.**
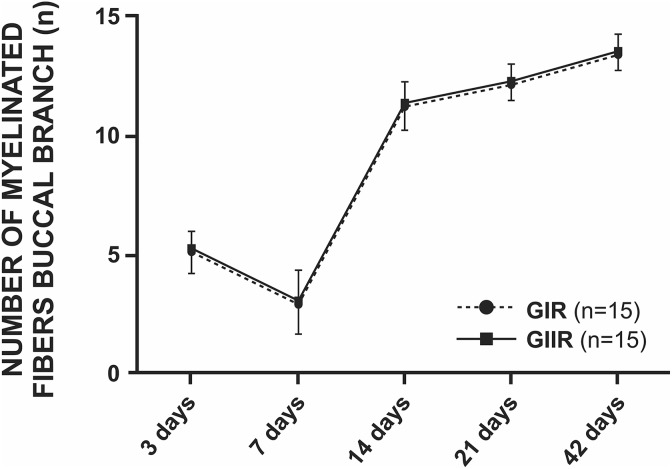
Statistical analysis of the number of myelinated fibers of the buccal branch. There was no statistical difference between groups.

Averages of the buccal branch did not present any difference in the studied parameters and periods between GIR and GIIR (Figs 9 and 10).

### NGF Expression

**Figure 11.**
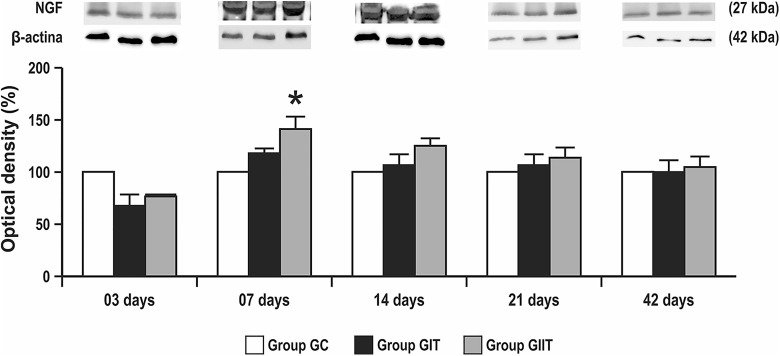
The expression levels of NGF to the facial nerve trunk for the GC, GI, and GII groups at all postoperative time. There were differences between the groups GII and GC at 7 postoperative days (*p < 0.05).

The expression of NGF at 7 postoperative days in GII was significantly higher than that of GC; an increase of 50% of optical density was observed, whereas there was no statistical significance between studied groups at 3, 14, 21, and 42 days after surgery. In addition, at 42 days, GI and GII exhibited a decrease in values of NGF protein expression, being close to the values expressed by GC (Fig. 11; *p < 0.05).

## Discussion

Functional assessment is fundamental in neural regeneration studies. Thus, a simplified observation scale was designed and adapted from other scales^33,34^ and it addressed the absence of movements, slight vibrissae tremor, and normal movement with posterior or anterior vibrissae positioning.

Some disagreements may arise when using simplified observation scales, as they present errors of subjectivity evaluation and low reproducibility by examiners due to the animals’ poor facial expression^35^. On the other hand, our results showed an overall value of Kappa (0.812) that can be attributed to the examiners’ previous training. In addition, it should be noted that the interval between the two measurements is enough time to reduce the influence of the examiner’s memory on a second measurement^36^.

Complete loss of movement or presence of minor tremor of vibrissae observed in animals are induced by denervation after traumatic nerve injury^37,38^. Thus, considering that effective movement and anterior positioning of vibrissae is a sign of complete functional recovery of the facial nerve^39^, it is evident that animals in GIIT presented complete functional recovery of vibrissae on the left hemiface at 14 days after surgery.

The fact that animals in GIIT presented better and earlier functional outcomes than GIT after 14 days is similar to the results described by other authors^13,33,35,38,40^. This can be explained by the neurogenic potential of DTSC populations influencing the mobilization of SC to the target tissue, and the secretion of neurotrophic factors that contribute to neuronal survival and provide a regenerative microenvironment that favors the recovery of muscle function^9,22,27,28^.

TEM analysis revealed that iDPSC influenced the axonal size in GIIT, since the mean outer and inner perimeters of axons were statistically greater in relation to GIT at all postoperative periods.

The outer and inner perimeters of myelinated axons in GIT and GIIT presented the same temporal recovery. At 14 days after surgery, there was reduction of axonal size coinciding with the process of Wallerian degeneration, which is completed in approximately 2 weeks. Between 21 and 42 postoperative days, the axons had gradual increase in dimensions, similar to the findings of other study^35^.

The thickness of the myelin sheath of axons in GIIT was statistically smaller than in GIT in all studied periods. However, the g-ratio of GIIT presented statistically higher values than GIT, corroborating the results described by other research^9^. In addition, the g-ratio of GIIT at 14 postoperative days showed a g-ratio value 0.59 ± 0.02, which was described for the facial nerve as ideal to maximize the nerve conduction rate^41^ and approaching an ideal value of 0.60 for peripheral nerves^30,42^, suggesting a good degree of remyelination.

Regarding the number of myelinated nerve fibers, even though GIIT showed smaller values compared with GIT, there was no significant difference between groups for all studied periods.

These results also indicate a difficult correlation between functional and histological improvement, as previously reported^43,44^. GIT presented more myelinated fibers, and GIIT presented better and earlier functional results concerning the recovery of vibrissae movements.

The qualitative results showed that GIIT presented histological improvement in comparison to GIT. These results can be explained by the increased macrophage concentration in the initial stages after injury (4–7 days), with removal of degenerating myelinic debris^45^, and by the presence of iDPSC modulating the effects of proinflammatory cytokines with increased levels of anti-inflammatory cytokines (IL-6 and IL-10) and a decrease in proinflammatory soluble factors such as IL-2, IL-4, TNF-α, and IFN-γ^46^. iDPSC also influence the migration and proliferation of SC into the target tissue, which in turn produce several types of trophic factors and receptors, including neurotrophins such as NGF, providing a favorable regenerative microenvironment^8,9,13,30^.

Nerve growth factor is recognized as a trophic molecule that is critical for the survival of sympathetic and sensory neurons. Nevertheless, other research^47^ has demonstrated that NGF prevented the apoptosis of motor neurons after induction of a facial nerve lesion through down-regulation of caspase-3 and PUMA expression (pro-apoptotic elements), suggesting a neuro-protective effect in injured motor neurons.

Our results showed NGF levels at 7 days with statistically higher values (*p* < 0.05) for GIIT compared with GC. At treatment completion, the NGF levels of GIT and GIIT decreased, and were close to the levels expressed by GC, corroborating the results described in other studies^13,14^ in which the NGF levels after injury are pronounced at 7 days, followed by a decrease at 21 days and near to the normal pattern after axonal regeneration.

The results observed for GIT and GIIT can be explained by the fact that crush lesions stimulate the migration of SC as they are associated with maintenance of the endoneurium; consequently, the NGF produced in the distal segment or target muscles can increase the expression levels of this neurotrophin at the lesion site, thus preventing facial nerve lesion-induced apoptosis^13,47^. Also, the presence of iDPSC at the lesion site may act as a paracrine communication triggered by secreted trophic factors and cytokines. These factors facilitate the migration, proliferation, and activation of SC^9^, and promote endogenous repair of neurologically damaged area, thus contributing to neuronal regeneration, regulating SC apoptosis and proliferation, leading to functional recovery^30,47^.

The blood–nerve barrier maintains the stability of the endoneural microenvironment such that endoneural vessels are not permeable under normal conditions^48^. In crush injury, a breakdown of the blood–nerve barrier happens and the vascular permeability increases, inducing plasmaprotein expression and contributing to axonal regeneration, although endoneural blood vessels recover in 5 days^49^.

In addition, the results of TEM analysis for buccal branch revealed similarities between groups GIR and GIIR, and no qualitative or quantitative differences were observed for all parameters after 42 days.

Based on this, our results suggest that iDPCS contribute to peripheral nerve regeneration through a favorable microenvironment for neural cell survival and migration of endogenous cells to the injured site. Moreover, the effects are probably local, as better results were obtained at the location of application of dental pulp stem cells.

## Conclusions

Stem cells potentiate regenerative medicine by their capability of differentiation into multiple lineage cells, and there are many stem cell sources for the repair and regeneration of injured peripheral nerves. Based on our experimental evidence, a single application of iDPSC immediately after facial nerve crush injury in rats can promote a positive local effect on neuro-protection, remyelination in 2 weeks of treatment, and may represent a novel therapeutic target to support regeneration after peripheral nerve injury and repair.

## Supplemental Material

Supplementary_Material - Rat Facial Nerve Regeneration with Human Immature Dental Pulp Stem Cells Click here for additional data file. Supplementary_Material for Rat Facial Nerve Regeneration with Human Immature Dental Pulp Stem Cells by Daniel Martinez Saez, Robson Tetsuo Sasaki, Daniel de Oliveira Martins, Marucia Chacur, Irina Kerkis and Marcelo Cavenaghi Pereira da Silva in Cell Transplantation
