# Supplementary material for: Rat Facial Nerve Regeneration with Human Immature Dental Pulp Stem Cells
Source: Cell Transplant. 2019 Aug 28;28(12):1573–84. doi: 10.1177/0963689719854446 (PMC6923557; doi:10.1177/0963689719854446)
Supplement: Supplementary_Material - Rat Facial Nerve Regeneration with Human Immature Dental Pulp Stem Cells [file Supplementary_Material.pdf]

**Supplementary Material I –** Mean values of number of myelinated fibers and g-ratio of timespan and studied groups.

| | | Facial Nerve Trunk (n=30) | | | | Buccal Branch (n=30) | | | |
|---|---|---|---|---|---|---|---|---|---|
| | | GIT (n=15) | | GIIT (n=15) | | GIR (n=15) | | GIIR (n=15) | |
| | | M | SD | M | SD | M | SD | M | SD |
| NUMBER OF MYELINATED FIBERS | 03 Days (n=3) | 7,15 | 1,02 | 4,99 | 0,57 | 5,13 | 0,50 | 5,23 | 0,47 |
| | 07 Days (n=3) | 4,94 | 1,05 | 2,79 | 0,40 | 2,92 | 0,59 | 3,03 | 0,66 |
| | 14 Days (n=3) | 13,29 | 0,72 | 11,13 | 1,07 | 11,27 | 1,14 | 11,37 | 1,13 |
| | 21 Days (n=3) | 14,21 | 0,94 | 12,05 | 0,70 | 12,19 | 0,72 | 12,29 | 0,71 |
| | 42 Days (n=3) | 15,47 | 1,04 | 13,32 | 0,72 | 13,46 | 0,71 | 13,56 | 0,72 |
| G-RATIO | 03 Days (n=3) | 0,36 | 0,02 | 0,43 | 0,02 | 0,37 | 0,02 | 0,39 | 0,01 |
| | 07 Days (n=3) | 0,33 | 0,02 | 0,40 | 0,02 | 0,35 | 0,02 | 0,36 | 0,02 |
| | 14 Days (n=3) | 0,52 | 0,01 | 0,59 | 0,02 | 0,54 | 0,02 | 0,55 | 0,02 |
| | 21 Days (n=3) | 0,46 | 0,01 | 0,54 | 0,01 | 0,48 | 0,01 | 0,50 | 0,01 |
| | 42 Days (n=3) | 0,42 | 0,01 | 0,49 | 0,01 | 0,44 | 0,01 | 0,45 | 0,01 |

M= Mean (estimated); SD= Standard Deviation (estimated)
